# Sol–gel synthesis of DyCrO_3_ and 10% Fe-doped DyCrO_3_ nanoparticles with enhanced photocatalytic hydrogen production abilities†

**DOI:** 10.1039/c8ra01585f

**Published:** 2018-04-17

**Authors:** Ragib Ahsan, Avijit Mitra, Saleh Omar, Md. Ziaur Rahman Khan, M. A. Basith

**Affiliations:** Department of Electrical and Electronic Engineering, Bangladesh University of Engineering and Technology Dhaka-1205 Bangladesh; Department of Physics, Bangladesh University of Engineering and Technology Dhaka-1000 Bangladesh mabasith@phy.buet.ac.bd

## Abstract

DyCrO_3_ and 10% Fe-doped DyCrO_3_ nanoparticles have been synthesized using a sol–gel method to investigate their performance in photocatalytic hydrogen production from water. The synthesized nanoparticles have been characterized by performing X-ray diffraction, energy dispersive X-ray spectroscopy and UV-visible spectrophotometric measurements. In addition, field emission scanning electron microscopy has been performed to observe their size and shape. The Fe-doped DyCrO_3_ nanoparticles show a significantly smaller band gap of 2.45 eV compared to the band gap of 2.82 eV shown by the DyCrO_3_ nanoparticles. The Fe-doped DyCrO_3_ nanoparticles show better photocatalytic activity in the degradation of rhodamine B (RhB) compared to the photocatalytic activity shown by both the DyCrO_3_ and Degussa P25 titania nanoparticles. The recycling and reuse of Fe-doped DyCrO_3_ four times for the photo-degradation of RhB shows that Fe-doped DyCrO_3_ is a stable and reusable photocatalyst. To evaluate the extent of the photocatalytic hydrogen production ability of the synthesized nanoparticles, a theoretical model has been developed to determine their “absorptance”, a measure of the ability to absorb photons. Finally, 10% Fe-doped DyCrO_3_ proves itself to be an efficient photocatalyst as it demonstrated three times greater hydrogen production than Degussa P25.

## Abstract 2

DyFe_0.1_Cr_0.9_O_3_ nanoparticles calcined at 700 °C demonstrate superior photocatalytic ability compared to that of DyCrO_3_ nanoparticles calcined at the same temperature.

## Graphical abstract

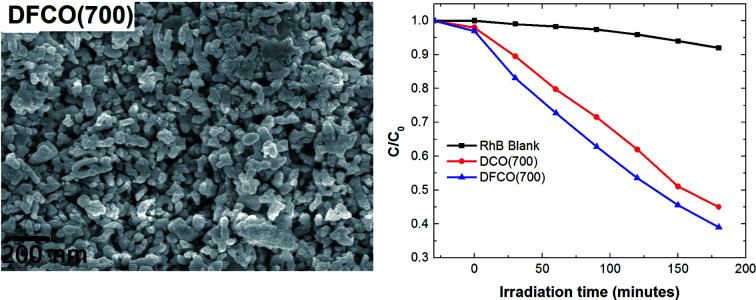

## 1 Introduction

Abundant sunlight on the surface of the earth can be considered a source of immense energy. However, if we want to utilize solar energy to suit our requirements, we have to convert it to more usable forms of energy such as heat, electricity *etc.* The converted energy also needs to be stored in a reproducible form to ensure its continuous availability even in the absence of sunlight. Nevertheless, low conversion efficiency and expensive storage have been hindering our journey towards unleashing the true potential of solar energy.^1–5^ Researchers have thus been motivated to undertake innovative initiatives to avoid these bottlenecks. The fabrication of complex optoelectronic devices such as multijunction and hot electron solar cells can ameliorate the efficiency problem by overcoming the Shockley–Queisser limit.^6–8^ While the theoretical limit of energy conversion efficiency for silicon solar cells is 29.1%,^9,10^ Yoshikawa *et al.* reported a 26.3% energy conversion efficiency for silicon heterojunction solar cells which is the highest efficiency reported to date.^11^ However, the implementation of such an architecture introduces additional fabrication expenses as it requires sophisticated substrates and precise fabrication techniques. Thus, it is desirable to devise a reasonably efficient conversion system with minimal fabrication costs and inexpensive storage.

By absorbing sunlight, substrateless photocatalyst powders dispersed in earth-abundant water can electrochemically split the water molecules to produce hydrogen. The absence of complex device architecture gives this technique an economic edge. In addition, the hydrogen gas produced is an environmentally friendly fuel that is easy and inexpensive to store. However, the efficiency of photocatalytic hydrogen production depends on certain abilities of the photocatalyst material. The generation of electron–hole pairs by absorbing solar photons and the inhibition of the recombination processes of the electron–hole pairs play major roles in determining the overall conversion efficiency.^13,23,24^ A material that possesses a band gap in the IR region can absorb photons over a broader range of the solar spectrum, which covers most of the solar energy that reaches the Earth’s surface.^12–14^ However, for successful electrochemical splitting of water, the potential of the conduction band minima (CBM) of the photocatalyst needs to be lower than the proton reduction potential (0 V *vs.* NHE, pH = 0) whereas the potential of the valence band maxima (VBM) needs to be greater than the oxidation potential of the hydroxyl ion (1.23 V *vs.* NHE, pH = 0).^15,16^ To sufficiently meet these conditions, the photocatalyst needs to have a minimum band gap of 1.23 eV or higher, depending on the potential of the CBM (*E*_cb_/e) and the VBM (*E*_vb_/e). Here, *E*_cb_ and *E*_vb_ are the energies of an electron at the CBM and VBM respectively. It is also necessary for the photocatalyst to have a high surface to volume ratio so that a large number of photons can be incident on its surface.^13,17,18^ Though there are several III–V and II–VI semiconductors such as gallium arsenide (GaAs), cadmium sulfide (CdS) *etc.* that meet the energy band gap and electrochemical conditions, they are not very stable under the photocatalytic reaction conditions.^19^ Nanoparticles of different metal oxides such as titanium dioxide (TiO_2_), bismuth ferrite (BiFeO_3_), lanthanum titanate (LaTiO_3_) *etc.*^15,17^ have been widely investigated since they usually satisfy most of the aforementioned conditions. Though nanoparticles tend to have wider band gaps than their bulk counterparts due to the quantum confinement effect, the metal oxide nanoparticles can be doped with appropriate foreign atoms to engineer redshifts in their band gaps.^20–22^ However, in many cases, a narrower band gap can cause *E*_cb_ to become positive and hinder the hydrogen production ability.^19^ Therefore, the pursuit of an efficient photocatalyst for hydrogen production from water requires careful engineering of both the band gap and *E*_cb_ of the metal oxides. Furthermore, non-centrosymmetric perovskite metal oxides possess spontaneous polarization as well as piezoelectric polarization.^25^ These different polarization mechanisms can spatially separate the electron and hole wavefunctions to reduce the probability of their recombination.^26^ Nanoparticles are inherently more strained than their bulk counterparts. This increased strain can make them more non-centrosymmetric by distorting their ideal unit cell structure and inducing a greater magnitude of polarization. Hence, inhibition of the electron–hole pair recombination processes should be more pronounced in perovskite metal oxide nanoparticles.

Dysprosium chromite (DyCrO_3_) is one such perovskite metal oxide that has the potential to be used in photocatalytic hydrogen production. DyCrO_3_ (DCO) has an orthorhombic crystal structure that belongs to the space group “*Pbnm*”.^27–29^ With a direct band gap of 2.8 eV, as reported in a previous investigation,^30^ DCO nanoparticles are capable of absorbing visible light close to the UV region of the solar spectrum. Remarkably, in addition to having a band gap that resides in the visible region, DCO has a negative *E*_cb_ and *E*_vb_ > 1.23 eV. Therefore, DCO can potentially be used as an efficient photocatalyst for hydrogen production if we can engineer a significant redshift in its band gap while maintaining a negative CBM potential. Earlier research on DCO nanoparticles demonstrated a redshift in the band gap when 30% of the Cr^3+^ ions were substituted with Fe^3+^ ions.^31^ However, the synthesis of 30% Fe-doped DCO nanoparticles is reported to be performed at a significantly higher calcination temperature^31^ compared to that of undoped DCO nanoparticles.^29^ For synthesizing nanoparticles, it is desirable to perform calcination at the lowest possible temperature so that the particle growth rate can be kept to a minimum. In addition, high-temperature processes are more expensive than the ones that are performed at lower temperatures. Moreover, large doping concentrations can cause significant distortions to the crystal structure of the parent material. However, as the Fe doping concentration is increased, *E*_cb_ tends to shift toward positive values due to the reduction in band gap. At a specific high doping concentration, *E*_cb_ may become positive rendering the photocatalyst incapable of producing hydrogen. Therefore, in this investigation, we have chosen to synthesize 10% Fe-doped DCO (DFCO) nanoparticles so that the calcination temperature can be kept closer to that of undoped DCO nanoparticles with minimum distortion of the parent crystal structure. Additionally, we aimed to obtain a considerable redshift in the band gap while keeping *E*_cb_ < 0. We synthesized both the DCO and DFCO nanoparticles using a sol–gel process^29,32^ and performed structural, morphological, elemental, and optical characterizations. A photocatalytic degradation test was performed on rhodamine B (RhB) dye using the DCO and DFCO nanoparticles as the photocatalyst. As observed from the experimental results, the DFCO nanoparticles caused considerably greater photocatalytic degradation than their undoped counterparts. Finally, the DFCO nanoparticles were subject to a photocatalytic hydrogen production experiment, and their performance in producing hydrogen was compared to that of a standard photocatalyst, Degussa P25 titania nanoparticles. The superior photocatalytic hydrogen production ability of DFCO nanoparticles was evident from the experimental result as we observed that the DFCO nanoparticles were capable of producing a substantially greater amount of hydrogen than the P25 nanoparticles.

## 2 Experimental section

### 2.1 Synthesis of DyCrO_3_ and DyFe_0.1_Cr_0.9_O_3_ nanoparticles

A citrate based sol–gel technique^29,32^ was adapted for the synthesis of DCO and DFCO nanoparticles. Dy(NO_3_)_3_·*x*H_2_O (Sigma Aldrich 99.9% pure), Cr(NO_3_)_3_·9H_2_O (Sigma Aldrich 99.9% pure), Fe(NO_3_)_3_·9H_2_O (Sigma Aldrich 99.9% pure), and citric acid (C_6_H_8_O_7_, Sigma Aldrich 99.5% pure) were used as precursors for synthesizing the DCO and DFCO nanoparticles. The ratio of citrate to Dy^3+^ ion was chosen to be 1 : 1. The precursors were stoichiometrically weighted and dissolved in 400 ml deionized water. The solution was magnetically stirred at room temperature and pressure (RTP) for 3 hours. The pH of the solution was then adjusted to around 9–10 by the dropwise addition of liquid ammonia (NH_4_OH). The solution was further magnetically stirred for 3 hours at RTP to obtain the homogeneous sol. This sol was then heated at 80 °C for 6 hours. After the evaporation of the solvent, a viscous gel was formed. The gel was further heated at a slightly elevated temperature to obtain the dried gel. The dried gel was calcined in air at 700 °C and 800 °C for 6 hours to finally obtain the DCO/DFCO nanoparticles. For brevity, the DCO and DFCO nanoparticles calcined at *x* °C will be referred to as DCO(*x*) and DFCO(*x*), respectively, from now on.

### 2.2 Characterization techniques

Structural characterization of the prepared samples was performed by obtaining their X-ray diffraction (XRD) patterns using a diffractometer (PANalytical Empyrean) with a Cu X-ray source (wavelength, *λ*: *K*_α1_ = 1.540598 Å and *K*_α2_ = 1.544426 Å). Rietveld refinement of the XRD patterns was performed using the FULLPROF package.^39^ Field emission scanning electron microscopy (FESEM) imaging of the samples was performed using a scanning electron microscope (XL30SFEG; Philips, Netherlands). The FESEM images were analyzed to determine the morphology of the nanoparticles. ImageJ software was used to analyze the size of the particles. An X-ray spectroscope attached to the same scanning electron microscope was used for performing energy-dispersive X-ray spectroscopy (EDS) of the samples for their elemental characterization. The samples were coated with platinum before they were subjected to FESEM and EDS measurements. A UV-visible spectrophotometer (UV-2600, Shimadzu) was used to obtain diffuse reflectance and absorbance spectra of the samples for wavelengths ranging from 200 to 1100 nm. There was a sample holder included in the package containing a thick layer of reference background material (Barium sulfate, BaSO_4_). The approximate size of the focused beam spot was 2 mm^2^. The sample nanoparticles were pressed by a glass rod on the reference material to ensure uniform thickness of the sample layer.^34^ After inserting the sample holder into the spectrophotometer, both the diffuse reflectance and absorbance spectra of the sample were measured.

### 2.3 Photocatalytic degradation test

A typical photocatalytic degradation test was performed on rhodamine B (RhB) dye^35,36^ using the synthesized nanoparticles as photocatalyst. After dissolving 10 mg RhB in 50 ml distilled water, 5 ml of the solution was extracted to measure its absorbance using a UV-vis spectrophotometer. The intensity of the absorbance peak (553 nm) was proportional to the amount of RhB present in the solution. The same method was used each time for determining the remaining amount of RhB in the solution. Then this solution was magnetically stirred in a dark room for 30 minutes to form a homogeneous solution. 40 mg of the photocatalyst sample along with 1 ml of 0.5 M HCl was then added to the solution. The solution was further stirred for 30 minutes to ensure an adsorption–desorption equilibrium between the sample nanoparticles and RhB in the solution. The suspension was then irradiated by a mercury–xenon lamp (Hamamatsu L8288, 500 W) to initiate the photocatalysis process. The irradiance values of the lamp were 6 mW cm^−2^ and 100 mW cm^−2^ in the UVA (315–400 nm) and visible (400–700 nm) region respectively. Prior to measuring the absorbance of this suspension, the nanoparticles were precipitated using a centrifuge machine at 5500 rpm for 10 minutes. The absorbance measurements were carried out at 30 minute intervals. To test the stability of the photocatalyst, the remaining photocatalyst nanoparticles in the suspension were separated by centrifugation after each round of photocatalytic degradation of RhB. In order to remove the residual RhB, the separated nanoparticles were washed with distilled water and dried afterward. These dried nanoparticles were then used as photocatalyst for the subsequent photocatalytic degradation cycle.

### 2.4 Photocatalytic hydrogen generation test

A photocatalytic hydrogen production test was performed in a slurry-type photochemical reactor following the typical procedures.^37,38^ 20 mg of the photocatalyst sample was dispersed into a 30 ml solution of deionized water (15 ml) and ethanol (15 ml). The suspension was magnetically stirred to obtain homogeneity, and the system was purged with argon gas for 30 minutes to provide the inert atmosphere required for the photocatalytic water-splitting process. By illuminating the solution with the same mercury–xenon lamp (Hamamatsu L8288, 500 W) used for the photocatalytic degradation test, the formation of gas over the suspension was observed. The generated gas was extracted at intervals of 30 minutes for 4 hours. The extracted gas was evaluated by a gas chromatography (GC) device equipped with a thermal conductivity detector (TCD) and a gas analyzer. The GC device was programmed for reverse polarization so that the hydrogen peaks could be obtained in the upward direction. The gas analyzer compared the peaks of the extracted gas to the peaks observed for standard hydrogen gas. The amount of generated hydrogen gas was determined in ml H_2_/g catalyst from this comparison.

## 3 Results and discussion

### 3.1 Structural characterization

**Fig. 1:**
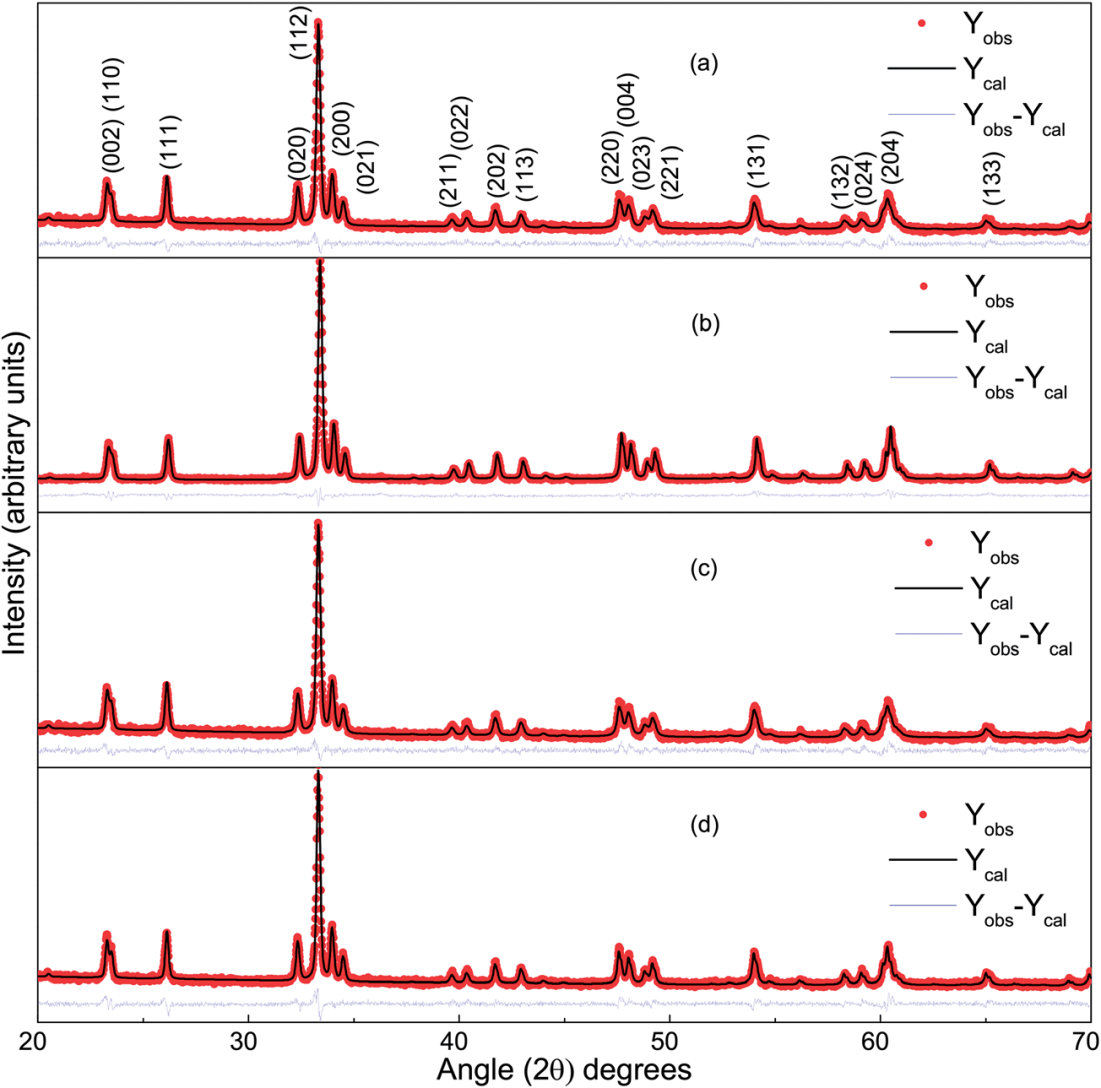
The Rietveld refined XRD pattern of (a) DCO(700), (b) DCO(800), (c) DFCO(700) and (d) DFCO(800) nanoparticles.

Fig. 1 presents the observed and Rietveld refined XRD patterns of DCO(700), DCO(800), DFCO(700) and DFCO(800) nanoparticles. The peak positions observed for all the samples match well with the peaks of single-phase orthorhombic perovskite DCO of the “*Pbnm*” space group (JCPDS card no. 251050). This result is consistent with the ones reported in previous investigations.^27–29^ As observed from Fig. 1, the peak positions of both DCO and DFCO nanoparticles are exactly the same. Since a crystal lattice is a periodic arrangement of the constituent ions, X-ray photons incident on the crystal see the lattice as a diffraction grating and generate a diffraction pattern with peaks at specific Bragg angles.^40,41^ DCO and DFCO nanoparticles have a very small difference in their crystal structure as the ionic radii of the Cr^3+^ (0.755 Å) and Fe^3+^ (0.785 Å) ions are nearly equal.^42^ The difference in ionic radii (0.03 Å) is negligible compared to the wavelength (1.5406 Å) of the X-ray. So, X-ray photons are diffracted by both structures in a very similar way, and nearly the same diffraction patterns are produced for both DCO and DFCO nanoparticles. In fact, even DyFeO_3_ produces the same XRD pattern as DCO as reported in an earlier investigation.^46^

Rietveld refinement of the powder XRD patterns suggests that all the samples are highly pure in phase as no other secondary non-perovskite phases can be detected. The precise control of the stoichiometry can be attributed to the successful suppression of impurity phases such as Fe, Fe_2_O_3_, Cr_2_O_3_, CrO_3_, DyCrO_4_*etc.* Lattice parameters of both the DCO and DFCO nanoparticles obtained from the refined patterns have been listed in Table 1.

**Table 1:** Lattice parameters *a*, *b* and *c* of DCO and DFCO nanoparticles synthesized at different calcination temperatures

Parameters	DCO(700)	DCO(800)	DFCO(700)	DFCO(800)
*a* (Å)	5.266	5.2643	5.2765	5.2773
*b* (Å)	5.5235	5.5208	5.535	5.5358
*c* (Å)	7.557	7.5537	7.5725	7.5709

Peak broadening of the XRD patterns is observed and can be primarily attributed to three factors: instrumental broadening, size broadening and strain broadening.^39–41^ Instrumental broadening needs to be corrected prior to extracting useful information from the observed patterns. The XRD pattern of a standard amorphous material, silicon, was obtained using the same diffractometer. This diffraction pattern can be considered as the transfer function of the specific instrument that we have used. The instrumental broadening corrected diffraction pattern is obtained by deconvoluting the transfer function of the instrument with the observed pattern of the corresponding sample.^39,43,44^ The integral breadth of the broadened peaks of the corrected patterns was then analyzed using the Scherrer equation^45^ and Williamson–Hall equation.^44^Table 2 lists the crystallite size and microstrain of the DCO(700), DCO(800), DFCO(700), and DFCO(800) nanoparticles. The crystallite sizes extracted using the Scherrer equation are on the nanoscale for each of the samples. The microstrain obtained using the Williamson–Hall equation is higher for the smaller nanoparticles which is consistent with the previous investigations.^39,47^

**Table 2:** Table of the average crystallite size and microstrain of the DCO and DFCO nanoparticles

Parameters	DCO(700)	DCO(800)	DFCO(700)	DFCO(800)
Average crystallite size (nm)	61	103	63	93
Microstrain (%)	0.001504	0.001437	0.0014563	0.000987

**Fig. 2:**
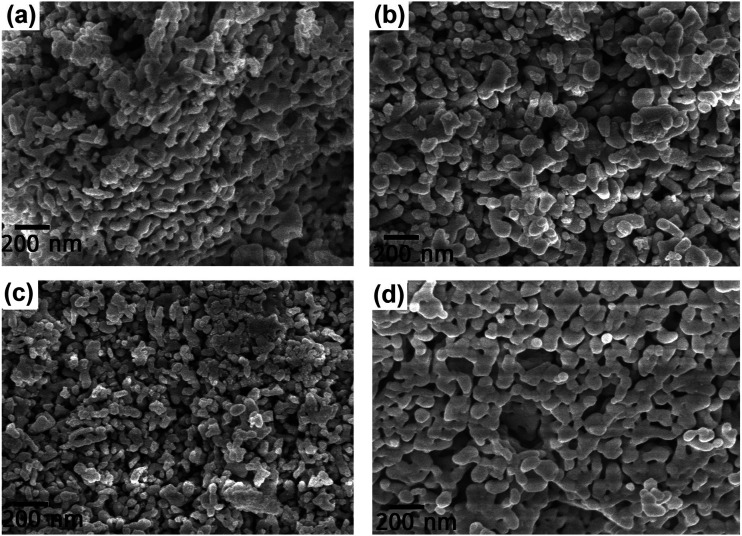
The FESEM images of (a) DCO(700), (b) DCO(800), (c) DFCO(700) and (d) DFCO(800) nanoparticles.

The FESEM images of the DCO and DFCO nanoparticles are shown in Fig. 2. As observed in Fig. 2(a–d), almost all of the particles have their dimensions on the nanoscale and are mostly irregular in shape rather than adhering to any specific shape. However, there is a considerable improvement in the shape of the DFCO(700) particles as most of them look nearly spherical with an average particle size of between 60 and 70 nm. For both the DCO(700) and DFCO(800) nanoparticles, a significant amount of agglomeration can be observed as the particles seem to be connected among themselves like a network. However, the agglomeration is considerably lower for the DFCO(700) nanoparticles. The agglomeration of the particles reduces the free energy of the system by reducing the total surface area. The nanoparticles synthesized at 800 °C have a greater particle size compared to the nanoparticles synthesized at 700 °C. This increase in particle size with elevated calcination temperature can be ascribed to the increased growth rate of the particles at higher temperatures.^33,48,49^ The smaller particle size of the DFCO(700) nanoparticles implies that they have a considerably larger surface to volume ratio compared to that of the DCO(700), DCO(800), and DFCO(800) nanoparticles which may be an important characteristic of an efficient photocatalyst.

### 3.2 Elemental characterization

ESI Fig. S1† shows the EDS spectra of the synthesized nanoparticles. For the DCO(700) and DCO(800) nanoparticles, the peaks of their EDS spectra suggest the presence of Dy, Cr and O atoms. In addition to the peaks of the Dy, Cr and O atoms, the EDS spectra of both the DFCO(700) and DFCO(800) nanoparticles have comparatively smaller peaks at 6.4 keV which confirms the presence of Fe atoms. The carbon content detected from each of these EDS spectra is very small. This can be attributed to the calcination process in air which removes almost all of the organic components of the gel. From Fig. S1,† a common peak for platinum can be observed in all of the EDS spectra which can be ascribed to the platinum coating of the particles prior to performing the spectroscopic analysis. Table 3 presents the elemental composition of the synthesized nanoparticles as analyzed from their EDS spectra.

**Table 3:** Table of the elemental composition of the DCO and DFCO nanoparticles

Elements (atom%)	DCO(700)	DCO(800)	DFCO(700)	DFCO(800)
Dy	19.71	19.97	19.84	20.08
Cr	18.33	18.69	17.78	18.57
Fe	0	0	2.07	2.22
O	61.96	61.34	60.31	59.13

### 3.3 Optical absorbance and band gap analysis

**Fig. 3:**
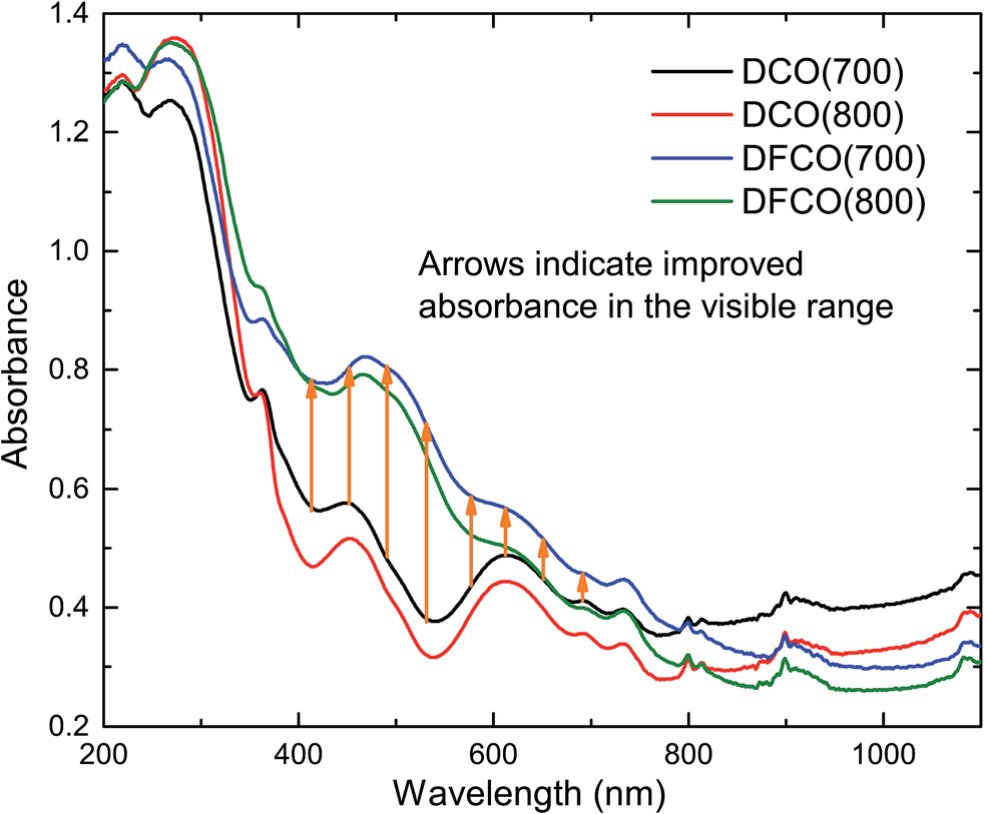
The DFCO nanoparticles calcined at 700 °C showing higher absorbance in the visible region than that of the DCO nanoparticles calcined at the same temperature.

The optical absorbance spectra of the DCO and DFCO nanoparticles are presented in Fig. 3. By extrapolating the linear region of an absorbance spectrum to the wavelength axis, we can determine the absorbance edge of the corresponding material. The absorbance edges of the DCO(700), DCO(800), DFCO(700) and DFCO(800) nanoparticles are found at 435, 473, 456 and 500 nm respectively. In addition, the DFCO nanoparticles show a higher absorbance than the DCO nanoparticles in the visible region. The diffuse reflectance spectra of the synthesized nanoparticles were also obtained to determine their optical band gap. We have used the modified Kubelka–Munk function for powder-form nanoparticles^34^ and Tauc’s law to convert the diffuse reflectance spectra to their corresponding Tauc plots. The linear region of the Tauc plot was then extrapolated to the energy axis and the energy axis intercept gave us the optical band gap of the corresponding material. The direct band gaps of the DCO(700), DCO(800), DFCO(700) and DFCO(800) nanoparticles are found to be 2.82, 2.72, 2.45 and 2.33 eV as shown in ESI Fig. S2.† An earlier investigation^30^ on DCO nanoparticles reported their band gap to be 2.8 eV which is consistent with our results. The DFCO nanoparticles show a significantly smaller optical band gap compared to the DCO nanoparticles. This redshift in the band gap implies that the DFCO nanoparticles can utilize the visible region of the solar spectrum more effectively than their undoped counterparts. As observed from Fig. S2,† the nanoparticles synthesized at a lower calcination temperature exhibit a higher band gap. This blueshift in the band gap can be attributed to the smaller average particle size of the nanoparticles synthesized at lower temperatures.^46,48^ As the particle size gets smaller, the quantum confinement effects are more prominent, and a blueshift in the band gap can be observed.^20,21^

When a material absorbs a photon with an energy greater than or equal to its band gap, an electron is elevated from its valence band to its conduction band, and an electron–hole pair is produced. The generated electron and hole should possess sufficient energy to perform the redox reactions that we are interested in *i.e.* the reduction of a proton and the oxidation of a hydroxyl ion. Therefore, we have also theoretically calculated the *E*_cb_ and *E*_vb_ of the synthesized nanoparticles (see ESI†). As observed from ESI Table S1,† both the DCO and DFCO nanoparticles have an *E*_cb_ < 0 which is necessary for reducing protons to hydrogen. However, DFCO(800) has a very small difference between the CBM potential (−0.1631 V) and the proton reduction potential (0 V) due to its smaller band gap. Therefore, the DFCO(800) nanoparticles may not be able to contribute to hydrogen production in reality. The VBM potential for both the DCO and DFCO nanoparticles is considerably above the oxidation potential of hydroxyl ions. Hence, the photogenerated holes are expected to readily oxidize the hydroxyl ions and produce oxygen.

### 3.4 Photocatalytic degradation activity and stability

**Fig. 4:**
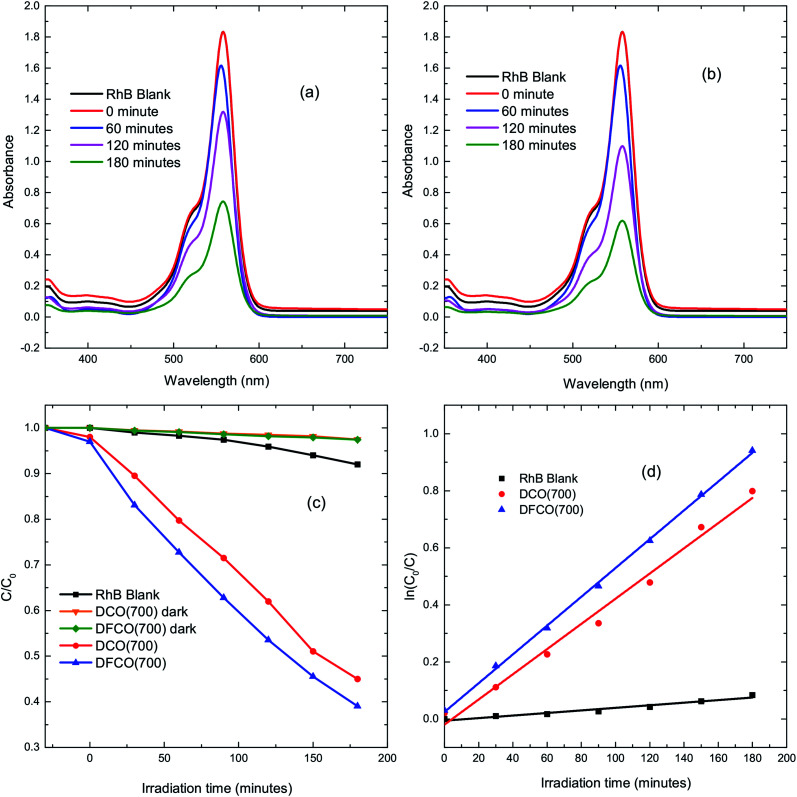
The UV-visible absorbance spectra for RhB solutions with (a) DCO(700) nanoparticles and (b) DFCO(700) nanoparticles at different time intervals. (c) The degradation of RhB *via* photocatalytic activity of the blank, DCO(700) and DFCO(700) sample with time. (d) The pseudo first-order kinetics fitted data for RhB photodegradation by the blank, DCO(700) and DFCO(700) samples.

The DCO(700) and DFCO(700) nanoparticles have been used as photocatalysts to perform the degradation of a standard dye, RhB, under UV-visible illumination. Fig. 4(a) and (b) show the absorbance spectra of RhB at different illumination times in the presence of the DCO(700) and DFCO(700) nanoparticles, respectively. Fig. 4(c) shows the cumulative photodegradation performance of the DCO(700) and DFCO(700) nanoparticles both in dark conditions and after 3 hours of illumination. There is no considerable photocatalytic degradation in dark conditions for either the DCO(700) or DFCO(700) nanoparticles. We believe that the adsorption of the RhB molecules to the catalyst nanoparticles is responsible for the slight degradation that occurs in dark conditions. On the other hand, the illuminated DCO(700) and DFCO(700) nanoparticles can degrade 53% and 62% of the RhB initially present in the solution respectively. Since the degradation due to the adsorption of RhB molecules is negligible, we can attribute the degradation by illuminated catalysts almost exclusively to photocatalytic degradation. The photodegradation reaction of RhB follows pseudo first-order reaction kinetics, and the degradation rate can be determined from the equation ln(*C*/*C*_0_) = −*kt*.^50^ Here, *k* is the degradation rate, and *C* and *C*_0_ are the concentrations of RhB at time *t* = *t* and *t* = 0 respectively. Fig. 4(d) shows the linear fit plots of ln(*C*_0_/*C*) *vs. t*. The slope of each straight line gives us the value of *k* for the corresponding photocatalyst. The blank RhB sample has a degradation rate of 4.63 × 10^−4^ min^−1^ compared to 4.08 × 10^−3^ min^−1^ and 5.37 × 10^−3^ min^−1^ for the DCO(700) and DFCO(700) nanoparticles respectively. With a 31% larger degradation rate, DFCO(700) can be considered to be a better photocatalyst than DCO(700). However, comparison with a standard photocatalyst such as Degussa P25 titania nanoparticles is also necessary to assess the photocatalytic performance of the DFCO(700) nanoparticles. As shown in a previous investigation by Esfahani *et al.*, P25 nanoparticles can degrade only 25% of the RhB dye initially present in the solution after 3 hours of UV-visible illumination which is equivalent to a degradation rate of 1.6 × 10^−3^ min^−1^.^51^ This degradation rate of the P25 nanoparticles is 235% smaller than that of the DFCO(700) nanoparticles. The higher surface to volume ratio and enhanced optical absorption in the visible region play important roles in activating the superior photocatalytic performance of the DFCO(700) nanoparticles. While a comparably smaller particle size helps them to achieve a higher surface to volume ratio, a narrower band gap enables them to absorb photons in the visible region of the solar spectrum where the irradiance of light is significantly higher than the UV region.^52,53^ Therefore, the DFCO(700) nanoparticles are capable of generating more electron–hole pairs that possess sufficient energy to commence photocatalytic degradation.

**Fig. 5:**
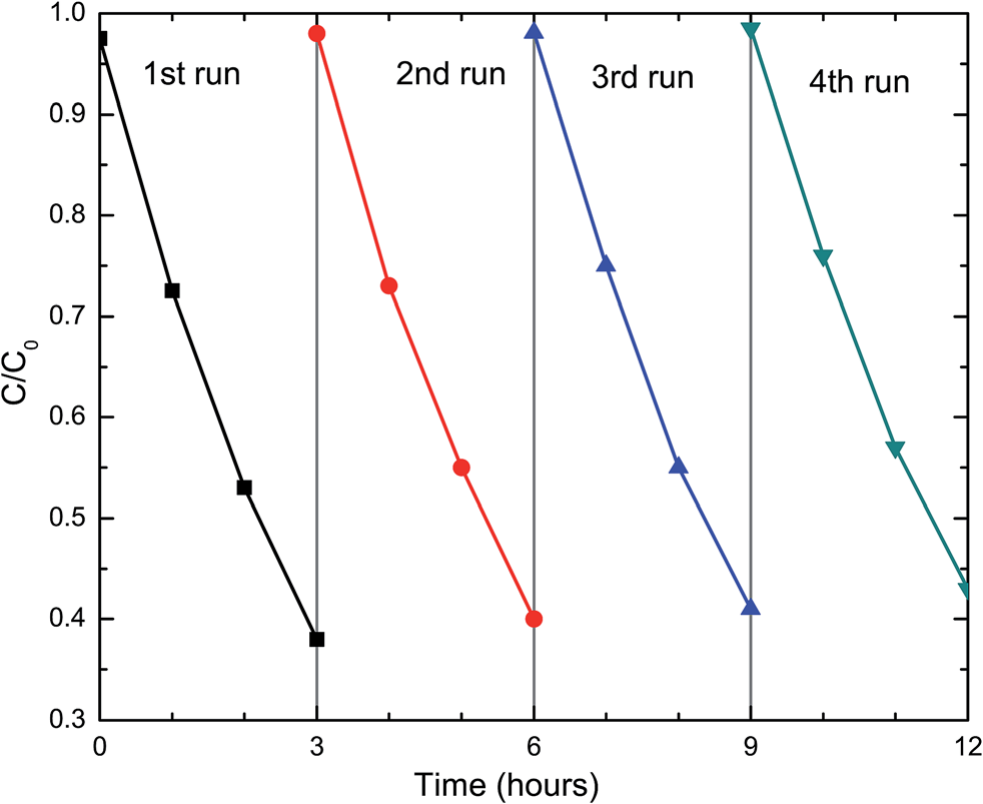
The recyclability of the DFCO(700) nanoparticles in four successive photocatalytic degradation experiments on RhB dye under UV-visible irradiation.

For practical applications, the photocatalysts need to be stable and reusable. The DFCO(700) nanoparticles were subjected to a recyclability test under the same test conditions. The test result, as observed in Fig. 5, implies that the DFCO(700) nanoparticles are stable after 4 cycles of photocatalytic degradation of RhB dye. However, the photocatalytic performance is slightly decreased after each successive cycle which is inevitable due to the loss of photocatalyst during the recycling process. Hence, we can consider the DFCO(700) nanoparticles to be stable and reusable for practical photocatalytic applications.

### 3.5 Photocatalytic mechanism

**Fig. 6:**
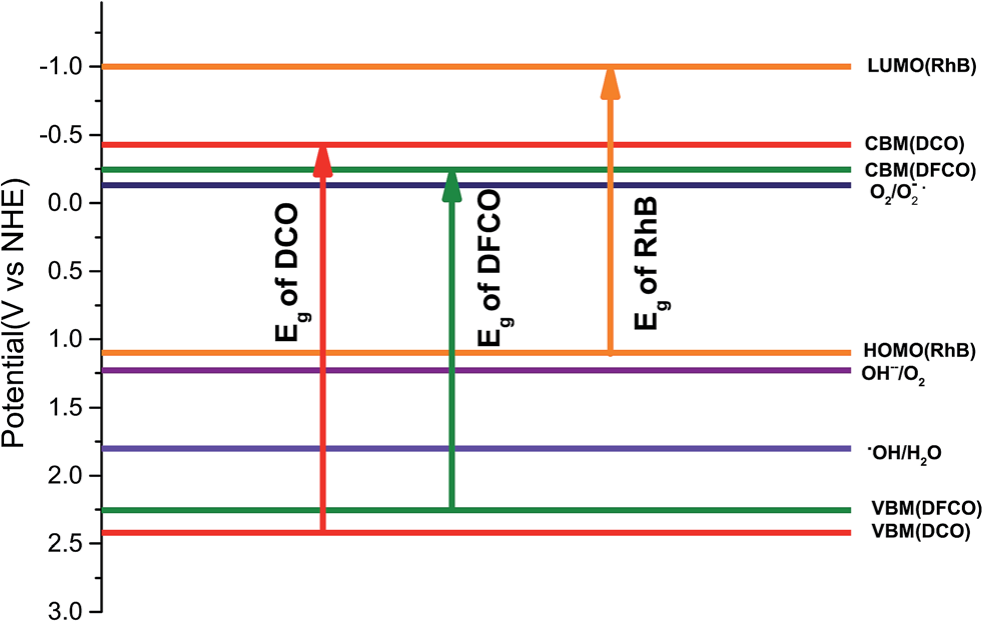
The energy band diagram for the DCO(700) and DFCO(700) nanoparticles.

Photodegradation of RhB dye is caused by certain redox reactions^54,55^ that can be catalyzed in the presence of DCO(700) and DFCO(700) nanoparticles. The photogenerated holes can react with the H_2_O molecules (redox potential of −0.13 V) to form ˙OH which causes the degradation of RhB. On the other hand, the photogenerated electrons can react with the surface adsorbed O_2_ (redox potential of 1.8 V) to produce O_2_^−^˙. This O_2_^−^˙ further reacts with RhB and causes degradation. Since both DCO(700) and DFCO(700) meet the conditions of *E*_cb_ < 1.8 eV and *E*_vb_ > −0.13 eV as shown in Fig. 6, the photogenerated electrons and holes are capable of performing the redox reactions required to degrade RhB. The redox reactions involved in the photodegradation of RhB can be summarized as follows.DyFe_*x*_Cr_1−*x*_O_3_ + *hν* → e^−^ + h^+^

1 h^+^ + H_2_O → ˙OH

2 e^−^ + O_2_ → O_2_^−^˙

h^+^ + RhB → photodegraded productsO_2_^−^˙ + RhB → photodegraded products˙OH + RhB → photodegraded products

The photogenerated electron–hole pairs also tend to recombine together to emit photons. It is desirable to suppress the recombination process since it inhibits photocatalytic activity by reducing the number of electron–hole pairs available for the redox reactions. The non-centrosymmetric structure of DCO(700) and DFCO(700) may induce spontaneous and piezoelectric polarization at the surface of the nanoparticles and spatially separate the electrons and holes to reduce the probability of recombination. However, further experiments such as time-resolved photoluminescence and photocurrent response analysis are required to elucidate the efficiency of the DCO(700) and DFCO(700) nanoparticles in suppressing the recombination process.

While generation of electron–hole pairs requires the absorption of photons with an energy ≥ *E*_g_, the absorption of sub-band gap photons can cause the photo-assisted oxidation of RhB under certain conditions. For photo-assisted oxidation of RhB dye, efficient electron transport needs to take place from the conduction band of the photocatalyst to the lowest unoccupied molecular orbital (LUMO) of RhB (−1 eV).^54^ However, this transport can only occur if *E*_cb_ < −1 eV. Since neither DCO(700) nor DFCO(700) can satisfy this condition (Fig. 6), the photo-assisted oxidation of the RhB dye is suppressed and sub-band gap photons do not contribute to the degradation process.

### 3.6 Absorptance model for evaluating photocatalyst performance in hydrogen production

Quantifying the response of different materials to the incident photons can provide an important insight into their comparative photocatalytic ability. Hence, we propose a mathematical model that can quantitatively compare different photocatalysts using their absorbance and diffuse reflectance spectra. Prior to performing the tedious photocatalytic hydrogen production experiment, such a model can be very helpful to rule out the relatively less efficient catalysts. When a photon is incident on the surface of a material, there are three possible outcomes: (a) absorption, (b) reflection and (c) transmission of the photon. An efficient photocatalyst is expected to absorb most of the photons incident on its surface. The ratio of the irradiance of the absorbed light (*I*_A_) to the irradiance of the incident light (*I*_0_) is defined as a quantity called “absorptance”. Absorptance can be considered as a quantitative measure of the efficiency of a photocatalyst in solar energy applications, *e.g.* solar hydrogen production. However, a photon is required to possess a minimum energy of 1.23 eV to contribute to the redox reactions responsible for splitting water into hydrogen and oxygen.^56,57^ Hence, it is useful for us to measure the absorptance of a photocatalyst for photons that have a minimum energy of 1.23 eV (corresponding to a wavelength of 1008 nm). However, we need to determine the irradiance of the incident and the absorbed light to calculate the absorptance. Since we aim to produce hydrogen using the sunlight that reaches the Earth’s surface, the spectrum of the solar energy evaluated at AM 1.5 (air mass coefficient) is essentially equal to the spectrum of the incident light. The AM 1.5 spectrum is a common standard for characterizing different solar cells and comparing their efficiency.^58–60^ Although we cannot directly measure the irradiance of the absorbed light using the spectrophotometer, we can measure the absorbance and diffuse reflectance spectra of the nanoparticles. Absorbance (*A*) is defined as the common logarithm of the ratio of the irradiance of the incident light (*I*_0_) to the irradiance of the transmitted light (*I*_T_) for a certain wavelength. Thus we can write, 
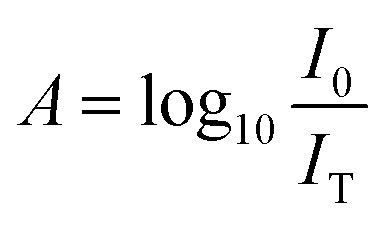
 and 
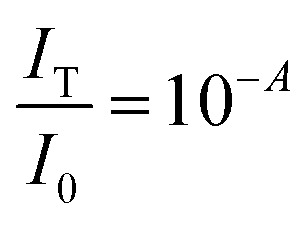
. In addition, the diffuse reflectance (*R*) is defined as the ratio of the irradiance of the diffuse reflected light (*I*_R_) to the irradiance of the incident light (*I*_0_) for a certain wavelength *i.e.*
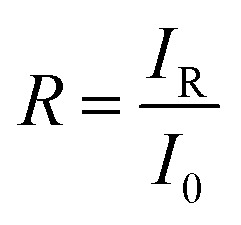
. Using the *I*_0_ values obtained from the AM 1.5 spectrum, we can determine *I*_T_ and *I*_R_ from their corresponding equations. We can now determine *I*_A_ for different wavelengths, from the equation *I*_A_ = *I*_0_ − *I*_R_ − *I*_T_, using the corresponding values of *I*_0_, *I*_T_ and *I*_R_. The area under the incident and the absorbed light spectra gives us the total irradiance of the incident and absorbed light over the range of wavelengths respectively. Therefore, we can now determine the absorptance from the ratio of the total irradiance of the absorbed light to the total irradiance of the incident light.

**Fig. 7:**
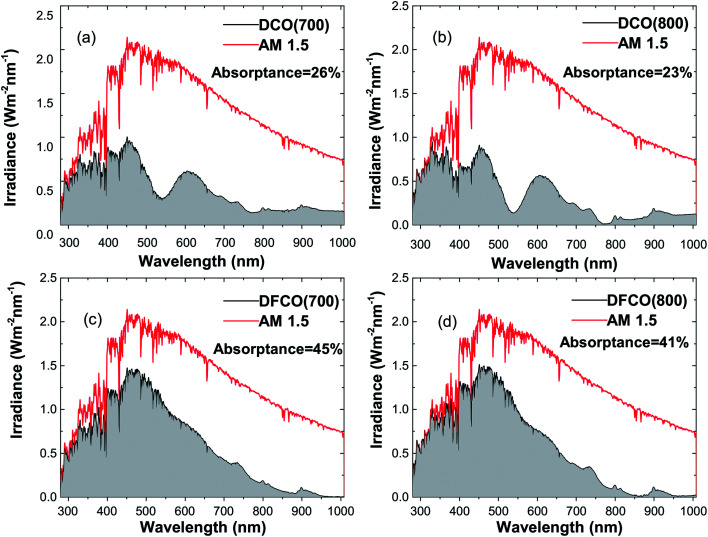
The absorbed light irradiance *vs.* wavelength plot in comparison with the AM 1.5 spectrum for (a) DCO(700), (b) DCO(800), (c) DFCO(700) and (d) DFCO(800) nanoparticles.

We have determined the absorptance of the synthesized nanoparticles from the AM 1.5 spectrum and their corresponding absorbance and diffuse reflectance spectra as presented in Fig. 7(a–d). It can be observed that DFCO(700) shows the highest absorptance of all of the samples at 45%.

### 3.7 Photocatalytic hydrogen production analysis

Since the DFCO(700) nanoparticles exhibit comparatively better photocatalytic degradation and absorptance than the DCO(700) nanoparticles, only the DFCO(700) nanoparticles were subjected to the photocatalytic hydrogen production test. In the absence of the DFCO(700) nanoparticles, there is no observable amount of hydrogen production. However, a significant amount of hydrogen production can be detected after the addition of the DFCO(700) nanoparticles. ESI Fig. S3† shows the amount of hydrogen produced (ml H_2_ g^−1^) with respect to the illumination time for the DFCO(700) nanoparticles. The photocatalytic hydrogen production of the DFCO(700) nanoparticles has also been compared to that of Degussa P25 nanoparticles under similar test conditions as shown in Fig. S3.†^61^ The comparison shows that the DFCO(700) nanoparticles can produce almost 200% more hydrogen than the P25 nanoparticles after 4 hours of illumination. The comparatively smaller band gap of the DFCO(700) nanoparticles (2.45 eV) than that of the P25 nanoparticles (3.1 eV) enables them to absorb more photons over a broader range of the solar spectrum. With an enhanced photon absorption ability, the DFCO(700) nanoparticles outperform the P25 nanoparticles in the generation of hydrogen. The considerably superior performance of DFCO(700) compared to that of a widely investigated standard photocatalyst asserts its potential in photocatalytic hydrogen production.

## 4 Conclusion

DyCrO_3_ and DyFe_0.1_Cr_0.9_O_3_ nanoparticles were synthesized using a sol–gel method by controlling different process parameters. We have performed a comparative investigation on the photocatalytic degradation and photocatalytic hydrogen production abilities of both undoped and Fe-doped DyCrO_3_ nanoparticles calcined at 700 °C. Achieving a redshift in the band gap, we have demonstrated the superior photon absorption ability of the Fe-doped nanoparticles calcined at 700 °C. While reducing the band gap, the band energies have also been engineered in such a way that the photogenerated electrons and holes are energetic enough to perform the redox reactions crucial to the photocatalysis process. With a smaller band gap, greater surface to volume ratio, and higher absorptance, the Fe-doped DyCrO_3_ nanoparticles calcined at 700 °C have proved their superiority over their undoped counterparts in the photocatalytic degradation of rhodamine B. These nanoparticles also exhibited 200% greater hydrogen production *via* water-splitting than a standard photocatalyst, Degussa P25.

## Conflicts of interest

There are no conflicts to declare.

## Supplementary Material

RA-008-C8RA01585F-s001
