# Supplementary material for: Sol–gel synthesis of DyCrO3 and 10% Fe-doped DyCrO3 nanoparticles with enhanced photocatalytic hydrogen production abilities
Source: RSC Adv. 2018 Apr 17;8(26):14258–67. doi: 10.1039/c8ra01585f (PMC9079860; doi:10.1039/c8ra01585f)
Supplement: RA-008-C8RA01585F-s001 [file RA-008-C8RA01585F-s001.pdf]

Electronic Supplementary Material (ESI) for RSC Advances.
This journal is © The Royal Society of Chemistry 2018

Journal Name

ARTICLE TYPE

Cite this: DOI: 10.1039/xxxxxxxxxx

Sol-gel synthesis of $DyCrO_3$ and 10% Fe-doped $DyCrO_3$ nanoparticles with enhanced photocatalytic hydrogen production abilities†

Ragib Ahsan,[ab] Avijit Mitra,[ab] Saleh Omar,[ab] Md. Ziaur Rahman Khan,[a] and M. A. Basith[b*]

[a] *Department of Electrical and Electronic Engineering, Bangladesh University of Engineering and Technology, Dhaka-1205, Bangladesh*

[b] *Department of Physics, Bangladesh University of Engineering and Technology, Dhaka-1000, Bangladesh. Email: mabasith@phy.buet.ac.bd*

† Electronic Supplementary Information (ESI) available: [details of any supplementary information available should be included here]. See DOI: 10.1039/b000000x/

Electronic supplementary information

EDS spectra

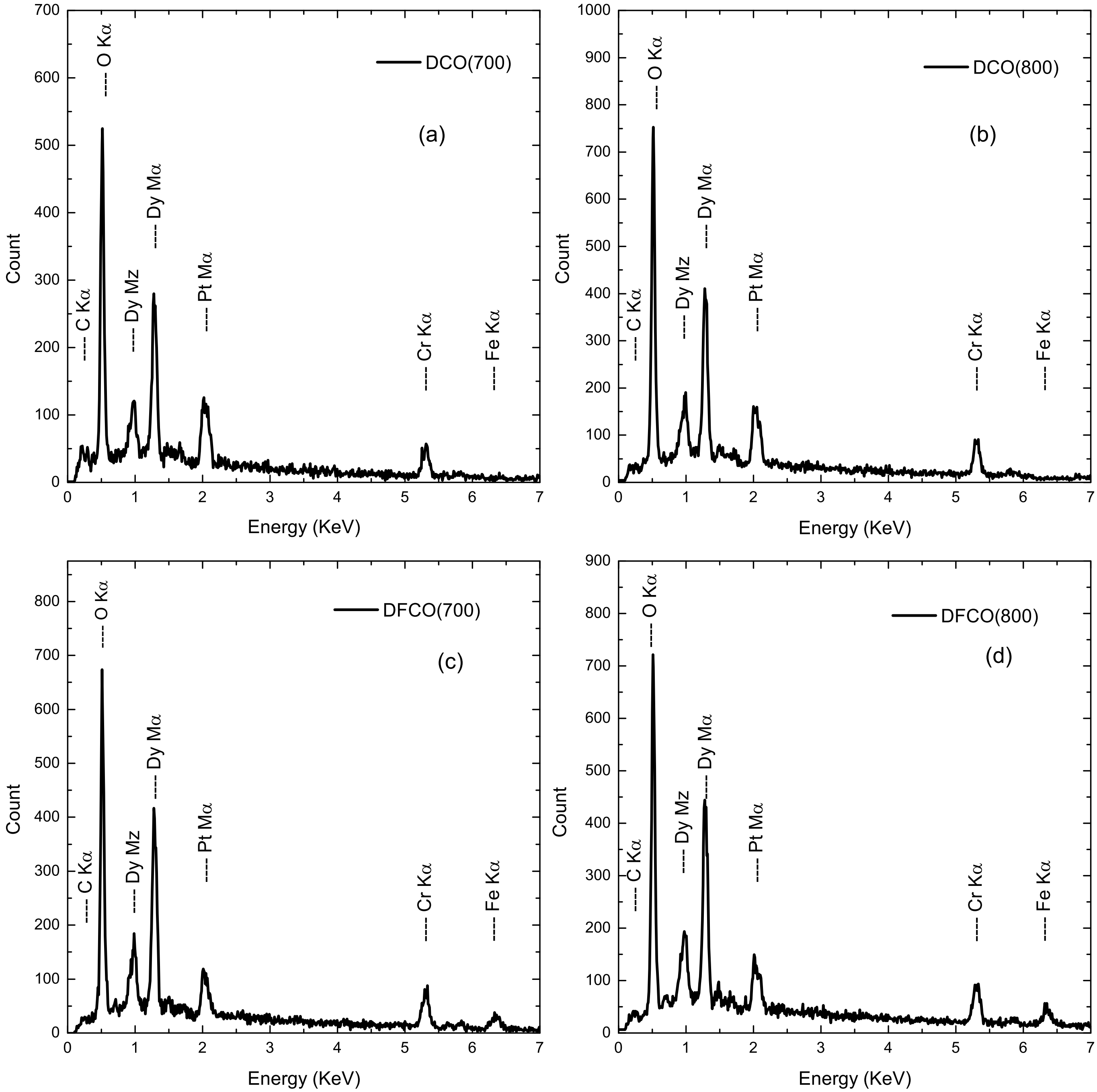

Fig. S1: EDS spectra of (a) DCO(700), (b) DCO(800), (c) DFCO(700), and (d) DFCO(800) nanoparticles

Calculation of CBM and VBM

Calculation of E_{cb} requires using the empirical formula[1]:

$$E_{cb} = \chi - E^{e} - 0.5E_{g} \quad (1)$$

Here, χ is the absolute electronegativity, E^{e} is the free electron energy on hydrogen scale (approximately 4.5 eV) and E_g is the band gap. For compound semiconductors, χ can be calculated from the geometric mean of the absolute electronegativity of the constituent

This journal is © The Royal Society of Chemistry [year]

atoms. Calculating E_{cb} with appropriate values of χ^{2} from equation (1), we can determine E_{vb}=E_{cb} + E_g. Table S1 lists these calculated values for DCO(700), DCO(800), DFCO(700), and DFCO(800) nanoparticles.

Table S1: Table of χ, E_g, E_{cb} and E_{cb} of DCO(700), DCO(800), DFCO(700), and DFCO(800) nanoparticles

Material	$\chi(eV)$	E_g (eV)	E_{cb} (eV)	E_{cb} (eV)
DCO(700)	5.4963	2.82	-0.4287	2.33213
DCO(800)	5.4963	2.72	-0.3787	2.38213
DFCO(700)	5.5048	2.45	-0.2452	2.2548
DFCO(800)	5.5048	2.33	-0.1631	2.1669

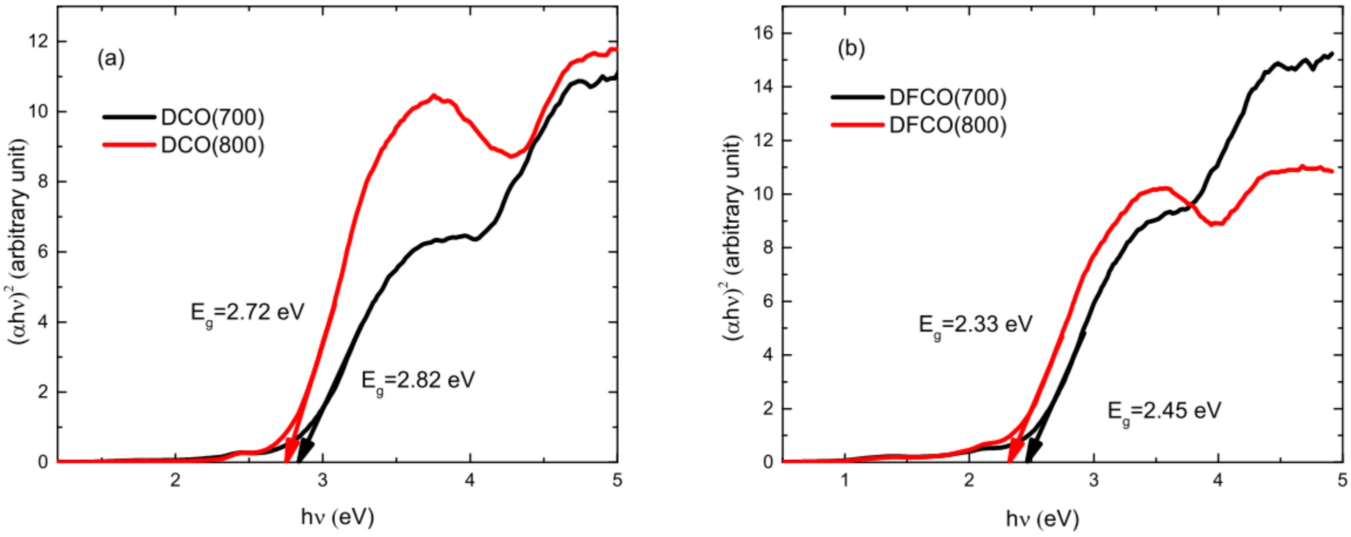

Fig. S2: Tauc plots obtained using modified Kubelka-Munk function from diffuse reflectance spectra for (a) DCO(700) and DCO(800), (b) DFCO(700) DFCO(800)

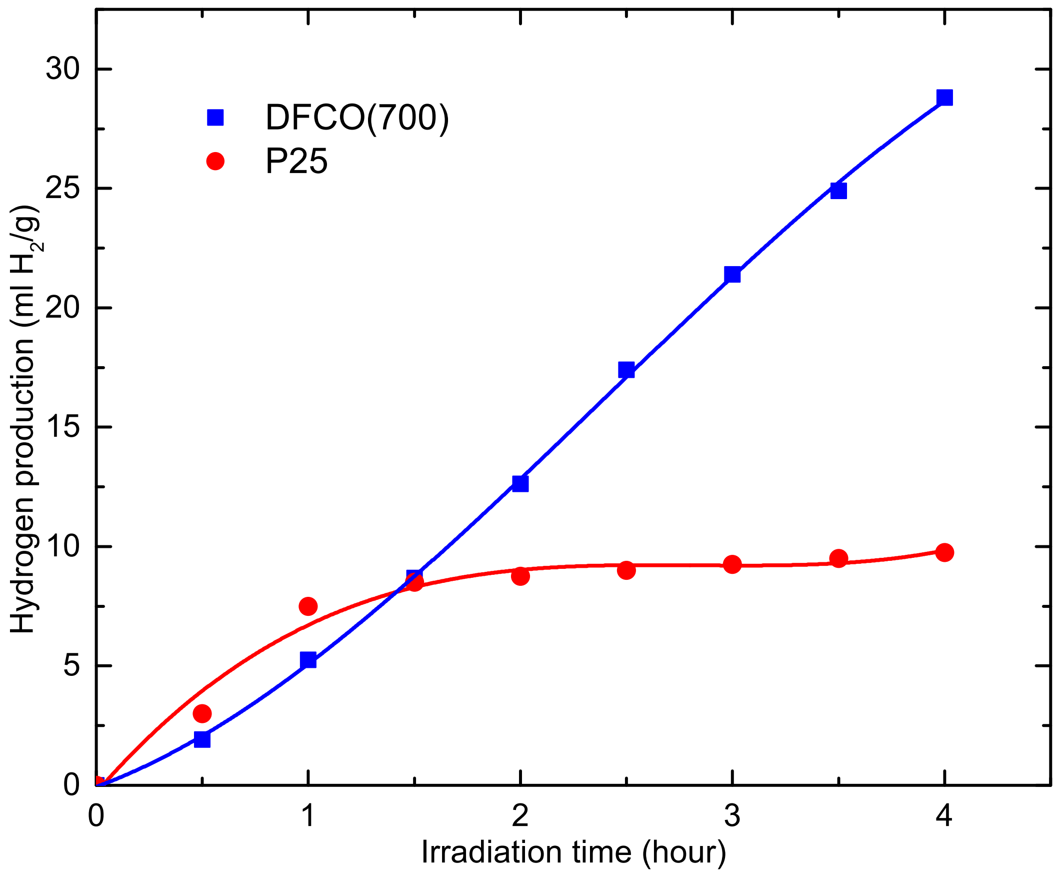

Fig. S3: Normalized photocatalytic hydrogen generation plotted against irradiation time for DFCO(700) and P25 nanoparticles

References

1 Y. Cui, S. M. Goldup and S. Dunn, RSC Advances, 2015, 5, 30372-30379.
2 R. G. Pearson, Inorganic chemistry, 1988, 27, 734-740.

This journal is © The Royal Society of Chemistry [year]
